# Genetic diagnosis and outcomes of intracytoplasmic sperm injection in South Chinese patients with congenital bilateral aplasia of the vas deferens

**DOI:** 10.1186/s12610-024-00233-2

**Published:** 2024-10-15

**Authors:** Haishan Hu, Qing Zhou, Yanlin Ma, Lingxiao Zhang

**Affiliations:** 1 Department of Reproductive Medicine, The First Affiliated Hospital of Hainan Medical University, Haikou, 570102 China; 2 Hainan Provincial Key Laboratory for human reproductive medicine and Genetic Research&Key Laboratory of Reproductive Health Diseases Research and Translation, Ministry of Education&Hainan Provincial Clinical Research Center for Thalassemia, The First Affiliated Hospital of Hainan Medical University, Hainan Medical University, Haikou, 571101 China; 3 Department of Cerebrovascular Diseases, Hainan Provincial Peoples Hospital, Haikou, 570102 China

**Keywords:** Congenital Bilateral Aplasia of the Vas Deferens (CBAVD), CFTR Mutations, ADGRG2 Mutations, Intracytoplasmic Sperm Injection (ICSI), Testicular Sperm Aspiration (TESA), Aplasie bilatérale congénitale du Canal déférent (CBAVD), Mutations du CFTR, Mutations de ADGRG2, Injection intracytoplasmique de spermatozoïdes (ICSI), Aspiration testiculaire des Spermatozoïdes (TESA)

## Abstract

**Background:**

Obstructive azoospermia commonly is caused by CBAVD(Congenital Bilateral Aplasia of the Vas Deferens), mainly due to the cystic fibrosis transmembrane conductance regulator (CFTR) and adhesion G protein-coupled receptor G2(ADGRG2) mutations. The genetic landscape for Chinese CBAVD patients is unclear, leading to debates over genetic screening, counseling, and assisted reproduction strategies. This study investigates the prevalence of CFTR and ADGRG2 mutations in a southern Chinese cohort of CBAVD patients and evaluates the impact of CFTR mutations on intracytoplasmic sperm injection (ICSI) outcomes.

**Results:**

CFTR mutations were identified in 71.4% (30/42) of CBAVD patients, with a total of 36 CFTR mutation sites across 13 types identified, including two novel mutations. A novel ADGRG2 mutation was also detected. Betweenthe CFTR mutation-CBAVD group and the non-CBAVD OA group, a significant difference was observed only in the 2 Pronuclei(2PN) rate (79.5% vs 86.2%, P = 0.0065), while fertilization rates, pregnancy rates, miscarriage rates, and live birth rates showed no significant differences. Between the CFTR mutation-CBAVD group and the CBAVD group without CFTR mutation, there were no significant differences in fertilization rates, 2PN rates, pregnancy rates, miscarriage rates, or live birth rates.

**Conclusion:**

Chinese CBAVD patients primarily exhibit mutations in the CFTR and ADGRG2 genes. Therefore, targeted gene testing for CFTR and ADGRG2 is more suitable compared to WES for CBAVD patients. Considering that the genetic factors of approximately 30% of CBAVD patients remain unknown, it is recommended to perform massive parallel sequencing for patients who test negative for CFTR and ADGRG2 gene screening. Despite these genetic factors, ICSI outcomes were not adversely affected, except for the 2PN rate. However, genetic counseling remains crucial for Chinese CBAVD patients before undergoing assisted reproduction.

## Résumé

**Contexte:**

L’azoospermie obstructive est généralement causée par une aplasie bilatérale congénitale du canal déférent (CBAVD), principalement due à des mutations du gène régulateur de la conductance transmembranaire de la mucoviscidose (CFTR) et à des mutations du récepteur G2 couplé aux protéines d’adhésion G (ADGRG2). Le paysage génétique des patients chinois atteints de CBAVD n’est pas clair, ce qui conduit à des débats sur le dépistage génétique, le conseil et les stratégies de procréation médicalement assistée. La présente étude examine la prévalence des mutations des gènes CFTR et ADGRG2 dans une cohorte de patients CBAVD du sud de la Chine ; elle évalue aussi l’impact des mutations CFTR sur les résultats de l’injection intracytoplasmique de spermatozoïdes (ICSI).

**Résultats:**

Des mutations du gène CFTR ont été identifiées chez 71,4 % (30/42) des patients atteints de CBAVD, avec un total de 36 sites de mutation CFTR dans 13 types identifiés, dont deux nouvelles mutations. Une nouvelle mutation du gène ADGRG2 a également été détectée. Entre le groupe mutation CFTR-CBAVD et le groupe d’azoospermie obstructive non CBAVD, une différence significative n’a été observée que pour le taux de 2 Pronuclei (2PN) (79,5 % vs 86,2 %, p = 0,0065), tandis que les taux de fécondation, les taux de grossesse, les taux de fausses couches et les taux de naissances vivantes n’ont montré aucune différence significative. Enfin, entre le groupe CBAVD avec mutation CFTR et le groupe CBAVD sans mutation CFTR, aucune différence significative n’est apparue dans les taux de fécondation, les taux de 2PN, les taux de grossesse, les taux de fausses couches ou les taux de naissances vivantes.

**Conclusions:**

Les patients chinois atteints de CBAVD présentent principalement des mutations localisées sur les gènes CFTR et ADGRG2. Par conséquent, les tests génétiques ciblés pour CFTR et ADGRG2 sont plus appropriés que le WES pour les patients CBAVD. Étant donné que les facteurs génétiques restent inconnus chez environ 30 % des patients atteints de CBAVD, il est recommandé d’effectuer un séquençage parallèle massif pour les patients dont le test de dépistage des gènes CFTR et ADGRG2 est négatif. Malgré ces facteurs génétiques, les résultats de l’ICSI n’ont pas été affectés négativement, à l’exception du taux de 2PN. Cependant, le conseil génétique reste crucial pour les patients chinois atteints de CBAVD avant de subir une procréation médicalement assistée.

## Introduction

Congenital Bilateral Aplasia of the Vas Deferens (CBAVD) is one of the significant factors leading to male infertility, accounting for 1% to 2% of male infertility cases and as high as 4% to 17% among men with azoospermia, reaching up to 25% in cases of obstructive azoospermia [1, 2]. Mutations in the cystic fibrosis transmembrane conductance regulator (CFTR) gene have been widely recognized as the primary cause of CBAVD, affecting the majority of CBAVD patients [3]. Studies have shown that the carriage rate of CFTR mutations in CBAVD patients is as high as 78% [4]. CFTR is a cAMP-dependent chloride ion channel protein present in the epithelial tissues of various organs, including the pancreas, intestines, sweat glands, and vas deferens. Dysfunction of this protein can lead to a range of clinical phenotypes, including cystic fibrosis, bronchiectasis, pancreatitis, and CBAVD [5, 6]. Mutations in CFTR can lead to dysfunction of the chloride ion channels on the cell membrane, preventing cells from regulating the flow of chloride ions and water molecules, thereby causing the reproductive tract to produce thick secretions that cannot be expelled, leading to the development of bilateral absence of the vas deferens [7, 8].

The CFTR gene is highly polymorphic, with mutations categorized into six types, ranging from Class I, which produces non-functional mRNA, to Class VI, characterized by unstable protein. These different types of mutations have varying impacts on the fertility of affected individuals. Particularly, Class I to III mutations are generally considered severe and may lead to cystic fibrosis, while Class IV to VI mutations are milder [9]. CBAVD is often seen as a mild clinical manifestation of cystic fibrosis, constituting a form of CF-related disease. In addition to the CFTR gene, the ADGRG2 gene variants, an X-linked adhesion G protein-coupled receptor G2, have also been found to be associated with CBAVD [10], adding complexity to the genetic background of this disease.

Assisted reproductive technology (ART), especially intracytoplasmic sperm injection (ICSI), offers a possibility of fertility for CBAVD patients [11]. However, there is a disagreement on the impact of CFTR gene mutations on the outcome of ART treatment. Some studies suggest that cystic fibrosis or CBAVD males carrying CFTR mutations may experience poorer ICSI treatment outcomes [11–13], while other research finds no significant difference in ICSI treatment results between CBAVD patients with or without CFTR gene mutations [14, 15].

Given that cystic fibrosis is a life-threatening disease, CFTR gene testing and counseling for CBAVD patients and their spouses are particularly important [16]. However, CFTR hotspot mutations are relatively rare in East Asian populations, leading to the possibility that existing screening strategies may not be applicable to the Chinese population [14, 15, 17, 18]. Therefore, research on the genetic background of CBAVD in the Chinese population is particularly critical.

This study included 42 CBAVD patients without classic CF symptoms, underwent whole-exome sequencing to screen for genetic variations. We compared the fertilization rate, 2PN rate, pregnancy rate, miscarriage rate, and live birth rate of CBAVD patients with CFTR gene mutations to non-CBAVD obstructive azoospermia patients from the same period. Additionally, we compared these outcomes between CBAVD patients with CFTR mutations and those without CFTR gene mutations after undergoing ICSI treatment. Through this study, we aim to provide more comprehensive clinical diagnosis, genetic counseling, and guidance on assisted reproductive technology for CBAVD, thereby reducing disagreements on the treatment efficacy of this condition and offering optimized treatment strategies for CBAVD patients.

## Materials and methods

### Study subjects

**Fig. 1:**
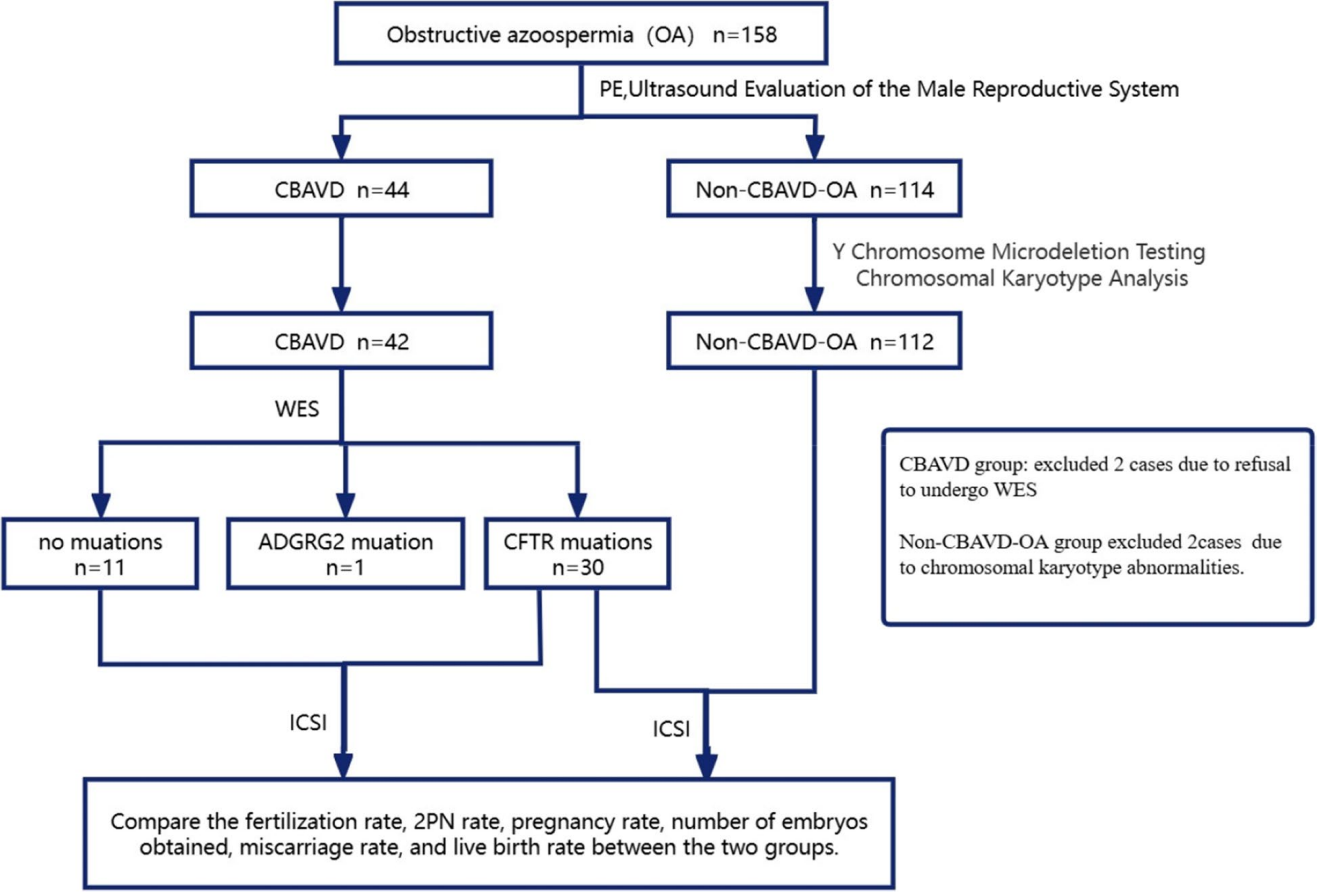
Flow chart for the study.design. Ultrasound Evaluation of the Male Reproductive System includes scrotal ultrasound and transrectal ultrasound. PE:Physical Examination.CBAVD: Congenital Bilateral Aplasia of the Vas Deferens;ICSI: Intracytoplasmic Sperm Injection; OA:obstructive azoospermia.CFTR: Cystic fibrosis transmembrane conductance regulator;ADGRG2: adhesion G protein-coupled receptor G2

This study retrospectively analyzed obstructive azoospermia patients who underwent ICSI cycles at our center from January 2020 to January 2023, most of whom were from South China and sought treatment for infertility. All participants underwent at least two semen collections spaced more than two weeks apart. Azoospermia was confirmed when examinations of centrifuged semen revealed no sperm, The semen analysis was conducted in strict accordance with the "World Health Organization Laboratory Manual for the Examination and Processing of Human Semen, Fifth Edition." [19].All obstructive azoospermia patients underwent medical history taking, physical examination,at least two semen parameter analyses, sex hormone tests, chromosomal analysis, and Y chromosome microdeletion testing, ultrasound examination of the male reproductive system (including scrotal ultrasound and transrectal ultrasound). A total of 158 obstructive azoospermia patients were included, Among them, 44 patients had obstructive azoospermia caused by CBAVD, of which 42 patients underwent whole-exome sequencing. There were 114 patients with non-CBAVD obstructive azoospermia, and all of these patients underwent ultrasound examination of the male reproductive system to exclude unilateral absence of the vas deferens or non-scrotal segment bilateral absence of the vas deferens. Among these, 2 cases underwent PGT-A due to chromosomal karyotype abnormalities.All study participants signed an informed consent form. We also tracked the ICSI outcomes of couples, including 42 CBAVD patients and 112 non-CBAVD patients, who entered the IVF cycle.including fertilization rates, 2PN rates, usable embryo numbers, pregnancy rates, miscarriage rates, and live birth rates. (Fig. 1).

### Sperm retrieval

Sperm were retrieved from all obstructive azoospermia patients using testicular sperm aspiration (TESA), performed by experienced andrologists. For TESA, the patient was placed in the supine position, followed by routine disinfection and local anesthesia with 1% lidocaine. Then, using a disposable 5 ml syringe, a small amount of testicular tissue was aspirated after puncturing the testis. The tissue was ground and pre-treated before observing the sperm count, motility, and morphology under an optical microscope.

### ICSI process

In this study, 42 CBAVD patients and 112 non-CBAVD obstructive azoospermia patients underwent controlled ovarian hyperstimulation. The daily FSH dose was adjusted based on the woman's oocytes, ovarian reserve, and various responses to ovarian stimulation. Triggering was done when at least one or two follicles reached a diameter of 18 mm, or more than three follicles reached a diameter of 17 mm. Oocytes were retrieved 36 h later, and sperm with relatively normal morphology were selected under 400 × magnification for ICSI. Embryos were graded according to the 2011 Istanbul consensus criteria for cleavage-stage embryo scoring [20], and high-quality embryos were selected for single embryo transfer on day three, with surplus high-quality embryos frozen for preservation. Serum β-HCG was measured 13 days after transfer, and when β-HCG ≥ 50U/L, luteal support treatment was continued. Clinical pregnancy was confirmed four weeks after transfer by the detection of a gestational sac via vaginal ultrasound. The outcomes of ICSI-ET (Intracytoplasmic Sperm Injection-Embryo Transfer) included fertilization rates, the number of usable embryos, clinical pregnancy rates, miscarriage rates, and live birth rates.

### Whole-exome sequencing

Genomic DNA (gDNA) was extracted from peripheral blood samples of each participant using a blood genomic DNA extraction kit (Tiangen Biotech, Beijing) following the manufacturer's instructions. The genomic DNA was then double-digested with two restriction enzymes and ligated to adapters. The ligation products were screened using AMPure XP beads to obtain fragments with specific sequence features and desired size. Libraries were prepared on the WES platform using the Illumina NovaSeq 6000 high-throughput sequencing technology. The output data for each sample was expected to be ≥ 50 M with Q30 > 80%. WES read data were aligned to the human reference genome hg19 using the BWA-MEM algorithm in the Sentieon toolkit v202010.02. The Sentieon toolkit, a set of software tools for analyzing genomic data obtained from DNA sequencing, was then used for sorting and indexing the aligned BAM files. Duplicate reads were removed, and base quality score recalibration (BQSR) was performed using Sentieon tools. Variant calling for each sample was done using Sentieon DNAscope's emit mode GVCF. Finally, joint variant calling for all samples was performed using the GVCFtyper algorithm. After Variant Quality Score Recalibration (VQSR), all variants were annotated and analyzed using the ANNOVAR tool. The pathogenicity and frequency of variants were annotated using the Human Gene Mutation Database (HGMD) and ClinVar. To ensure the authenticity of data analysis, only variants filtered as PASS by VQSR, with an Inbreeding Coeff less than -0.3, and filtering out AC0 variants (AC0: no samples have high-quality genotypes (depth >  = 10, genotype quality >  = 20, and for heterozygous genotypes, minor allele balance > 0.2)), referring to the gnomAD database filtering criteria. Variant confirmation and familial cosegregation analysis were conducted through Sanger sequencing.

### Statistical analysis

Statistical analysis was performed using SPSS software package version 24.0. Continuous variables were represented as median (25th percentile ~ 75th percentile) and analyzed using the Wilcoxon rank-sum test. Categorical variables were represented as percentages and compared using the chi-square test and the Fisher's precision probability test. A P-value < 0.05 was considered statistically significant.

## Results

This retrospective study included 154 obstructive azoospermia (OA) patients, Among them, 42 cases were caused by bilateral absence of the vas deferens and were classified into the CBAVD group. None of the patients diagnosed with CBAVD exhibited typical symptoms of cystic fibrosis. The remaining 112 cases were classified into the non-CBAVD OA group. Among the 112 non-CBAVD OA patients, 29 were primarily infertile and 83 were secondarily infertile. The main causes in this group were 54 cases of epididymitis, 22 cases post-epididymovasostomy, 9 cases post-vasovasostomy, 3 cases with a history of genital tuberculosis, 11 cases post-vasectomy, 5 cases due to inguinal and pelvic surgery, and 8 cases caused by trauma. There were no significant differences between the two groups in terms of age, semen abstinence time, FSH, LH, E2, PRL, and T levels. However, there were significant differences in ejaculate volume and pH value between the two groups. The baseline information is shown in Table 1.

**Table 1:** Baseline characteristics of the patients

Characteristic		No-CBAVD-OA (*n* = 112)	CBAVD (*n* = 42)	*P* value
Male age (years)		33(30 ~ 36)	31.5(30 ~ 33)	0.062
Types of infertility (n)	Primary infertility	29	42	
	Secondary infertility	83		
Etiology (n)	Epididymitis	54		
	Post-epididymovasostomy	22		
	Post-vasovasostomy	9		
	Genital tuberculosis	3		
	vasectomy	11		
	Inguinal or pelvic surgeries	5		
	trauma	8		
Semen parameters	Abstinence period	5(4 ~ 5.25)	5(3.25 ~ 5)	0.256
	Volume(mL)	3.2(2.7 ~ 4.0)	0.9(0.5 ~ 1.0)	< 0.000001
	pH	7.6(7.4 ~ 7.8)	6.9(6.6 ~ 7.2)	< 0.000001
Sex hormones	FSH(IU/L)	5.9(2.9 ~ 8.1)	5.5(3.9 ~ 8.1)	0.5
	LH(IU/L)	5.0(3.3 ~ 6.4)	4.7(3.5 ~ 5.8)	0.57
	T(nmol/L)	16.3(10.8 ~ 21.6)	15.8(13.6 ~ 21.5)	0.386
	E2(pmol/L)	99.6(72.8 ~ 121.0)	97.4(68.4 ~ 111.8)	0.218
	PRL(mIU/L)	174.5(117.4 ~ 235.1)	204.7(121.1 ~ 255.2)	0.31

Comparison of baseline characteristics between patients with Congenital Bilateral Aplasia of the Vas Deferens (CBAVD, *n* = 42) and obstructive azoospermia without CBAVD (No-CBAVD-OA, *n* = 112)

Continuous variables were presented as medians (first quartile-third quartile) and compared using the Mann–Whitney U rank-sum test

Categorical variables were presented as % (n/N) and compared using the chi-squared test

*P* value < 0.05 was considered statistically significant

*CBAVD* Congenital Bilateral Aplasia of the Vas Deferens, *OA* Obstructive azoospermia, *FSH* Follicle‑stimulating hormone, *LH* Luteinizing hormone, *E2* Estradiol, *T* Testosterone, *PRL* Prolactin

Through whole-exome sequencing, we identified that 30 of the 42 CBAVD patients (71.4%) carried at least one mutation in the CFTR gene, with a total of 36 CFTR mutation sites encompassing 13 different types identified. Among these mutations, six were classified as pathogenic, three as likely pathogenic, and four as variants of uncertain significance. Within these 30 cases, seven patients exhibited homozygous mutations of the CFTR gene, and six had compound heterozygous mutations. Among the 36 CFTR mutation sites carried by patients, 14 types (38.9%, 14/36) were missense mutations, 21 (58.3%, 21/36) were splice mutations, and one (2.8%, 1/36) was a nonsense mutation. Two novel CFTR mutations were identified, one missense mutation p.Ile1023Arg and one nonsense mutation c.2125C > T. Additionally, a novel ADGRG2 missense mutation (c.473G > A, p.Arg158His) was identified, classified as a variant of uncertain significance (Table 2).

**Table 2:** Genetic variants were detected in the 42 CBAVD patients

Patient	Gene	Zygosity	ACMG class	DNA change	Protein change	Mutation type	Exon/Intron
P1	CFTR	Heterozygous	P	C.1210-12T[5]		Splicing	Intron9
P2	CFTR	Homozygous	P	C.1210-12T [5]		Splicing	Intron9
P3	CFTR	Homozygous	P	C.1210-12T [5]/c.1210-34TG		Splicing	Intron9
P4	CFTR	Homozygous	P	C.1210-12T[5]		Splicing	Intron9
P5	CFTR	Heterozygous	P	C.1210-12T[5]		Splicing	Intron9
	CFTR	Heterozygous	P	C.2125C >T		Nonsense	Exon14
P6	CFTR	Homozygous	P	C.1210-12T[5]		Splicing	Intron9
P7	CFTR	Heterozygous	P	C.1210-12T[5]		Splicing	Intron9
P8	CFTR	Heterozygous	LP	c.1210-11T > G		Splicing	Intron9
P9	CFTR	Homozygous	P	C.1210-12T[5]		Splicing	Intron9
P10	CFTR	Heterozygous	P	C.1210-12T[5]/c.1210-34TG		Splicing	Intron9
P11	CFTR	Heterozygous	P	c.2909G > A	p.Gly970Asp	Missense	Exon18
P12	CFTR	Heterozygous	P	C.1210-12T[5]/c.1210-34TG		Splicing	Intron9
		Heterozygous	P	c.3068 T > G	p.lle1023Arg	Missense	Exon19
P13	CFTR	Heterozygous	VUS	c.2042A > T	p.Glu681Val	Missense	Exon 14
P14	CFTR	Heterozygous	P	c.2909G > A	p.Gly970Asp	Missense	Exon18
P15	CFTR	Heterozygous	LP	c.1407G > T	p.Met469Ile	Missense	Exon 11
P16	CFTR	Heterozygous	LP	c.4262 T > A	p.Met469Ile	Missense	Intron 27
P17	CFTR	Heterozygous	P	C.1210-12T[5]		Splicing	Intron9
P18	CFTR	Homozygous	p	C.1210-12T[5]		Splicing	Intron9
P19	CFTR	Heterozygous	P	C.1210-12T[5]		Splicing	Intron9
		Heterozygous	P	C.2909G > A	p.Gly970Asp	Missense	Exon18
P20	CFTR	Heterozygous	LP	c.4056G > C	p.Q1352H	Missense	Exon14
		Heterozygous	VUS	c.1210-11 T > G		Splicing	Intron9
P21	CFTR	Heterozygous	VUS	c.2042A > T	p.Glu681Val	Missense	Exon 14
P22	CFTR	Heterozygous	P	c.350G > A	p.Arg117His	Missense	Exon7
		Heterozygous	LP	c.4056G > C	p.Gln1352His	Missense	Exon25
P23	CFTR	Heterozygous	P	C.1210-12T[5]		Splicing	Intron9
P24	CFTR	Heterozygous	VUS	c.1210-11T > G		Splicing	Intron9
P25	CFTR	Heterozygous	P	C.1210-12T[5]/c.1210-34TG		Splicing	Intron9
P26	CFTR	Heterozygous	LP	c.4056G > C	p.Gln1352His	Missense	Exon25
		Heterozygous	VUS	c.601G > A	p.Val201Met	Missense	Exon6
P27	CFTR	Heterozygous	VUS	c.1666A > G	p.lle556Val	Missense	Exon12
P28	CFTR	Heterozygous	P	C.1210-12T[5]		Splicing	Intron9
P29	CFTR	Homozygous	P	C.1210-12T[5]		Splicing	Intron9
P30	CFTR	Heterozygous	P	C.1210-12T[5]		Splicing	Intron9
P31	ADGRG2	Semizygote	VUS	c.473G > A	p.Arg158His	Missense	Exon12

Genetic Variants in CBAVD Patients: Whole exome sequencing of 42 CBAVD patients revealed that 30 patients had CFTR mutations and one patient had an ADGRG2 mutation. The table categorizes patients (P1-P31) based on the detected gene mutations, their zygosity, ACMG classification, DNA changes, protein changes, mutation types, and the affected exon or intron. *CFTR* Cystic fibrosis transmembrane conductance regulator, *ADGRG2* Adhesion G protein-coupled receptor G2, *P* Pathogenic, *LP*, Likely pathogenic mutations, *VUS* Variants of uncertain significance

In the analysis of ICSI cycles, this study included 154 first ICSI cycles, all using fresh testicular sperm. Initially, we performed a comparative analysis of the ICSI outcomes between 30 cycles of CBAVD patients with CFTR gene mutations and 112 cycles of non-CBAVD obstructive azoospermia patients. Record the fertilization rate, 2PN rate, and the number of usable embryos in the embryology lab. Additionally, document the clinical outcomes of the first transfer of day 3 embryos, including pregnancy rate, miscarriage rate, and live birth rate.After comparing the female age, male age, fertilization rate, 2PN formation rate, pregnancy rate, miscarriage rate, and live birth rate between the two groups, no statistical differences were found in female age, male age, fertilization rate, pregnancy rate, miscarriage rate, and live birth rate. However, the 2PN formation rate in the CBAVD group with CFTR gene mutations was 79.52% (233/293), significantly lower than 86.15% (970/1126) in the non-CBAVD obstructive azoospermia group, with a P-value of 0.0065(Table 3).

**Table 3:** Laboratory and clinical outcomes of OA couples in ICSI cycles

	CFTR Mutation	no-CBAVD-OA	*P*
n	30	112	
Male age (years)	32(30 ~ 33)	33(30 ~ 36)	0.145
Female age (years)	29(27.25 ~ 33)	31(27.75 ~ 35)	0.171
Fertilization rate	77.10% (293/380)	76.76% (1126/1467)	0.94
2PN rate	79.52% (233/293)	86.15% (970/1126)	0.0065
Average number of available embryos	5(3 ~ 6.75)	4(2 ~ 7)	0.212
Clinical pregnancy rate/fresh ET (n)	60% (18/30)	52.68% (59/112)	0.475
Miscarriage Rate	16.7% (3/18)	11.8% (7/59)	0.897
Live Birth Rate	50% (15/30)	46.43% (52/112)	0.728

This table compares the laboratory and clinical outcomes of couples with Congenital Bilateral Aplasia of the Vas Deferens (CBAVD) and those with other forms of obstructive azoospermia (No-CBAVD-OA) undergoing ICSI cycles

Continuous variables were presented as medians (first quartile-third quartile) and compared using the Mann–Whitney U rank-sum test

Categorical variables were presented as % (n/N) and compared using the chi-square test

*CFTR* Cystic fibrosis transmembrane conductance regulator, *2PN rate* 2 Pronuclei rate, *CBAVD* Congenital bilateral absence of the vas deferens, *ICSI* Intracytoplasmic Sperm Injection, *OA* Obstructive azoospermia

*P* value < 0.05 was considered statistically significant

Meanwhile, to further analyze whether the CFTR gene affects ICSI outcomes, we divided the 42 CBAVD patients into two groups: one group of 30 CBAVD patients with CFTR mutations and another group of 12 CBAVD patients without CFTR mutations, excluding one case due to the presence of an ADGRG2 mutation. We then compared the two groups regarding female age, male age, fertilization rate, 2PN formation rate, pregnancy rate, miscarriage rate, and live birth rate. The results showed no statistically significant differences between the two groups in terms of female age, male age, fertilization rate, 2PN formation rate, pregnancy rate, miscarriage rate, and live birth rate (Table 4).

**Table 4:** Laboratory and Clinical Outcomes of CBAVD Couples with and without CFTR Mutation in ICSI Cycles

Characteristic	CFTR Mutation	no- CFTR Mutation	*P*
n	30	11	
Male age (years)	32(30 ~ 33)	31(27 ~ 33)	0.145
Female age (years)	29(27.2 ~ 33)	30(28 ~ 34)	0.171
Fertilization rate	77.1% (293/380)	72.4% (92/127)	0.28
2PN rate	79.5% (233/293)	77.2% (71/92)	0.66
Average number of available embryos	5(3 ~ 6.75)	3(1.5 ~ 6.5)	0.18
Clinical pregnancy rate/fresh ET (n)	60% (18/30)	63.6% (7/11)	1
Miscarriage Rate	16.7% (3/18)	14.2% (1/7)	1
Live Birth Rate	50% (15/30)	54.5% (52/112)	1

This table compares the laboratory and clinical outcomes of couples with CBAVD undergoing ICSI cycles, divided into groups based on the presence or absence of CFTR mutations

Categorical variables were presented as % (n/N) and compared using the Fisher's precision probability test

Continuous variables were presented as medians (first quartile-third quartile) and compared using the Mann–Whitney U rank-sum test

*CFTR* Cystic fibrosis transmembrane conductance regulator, *2PN rate* 2 Pronuclei rate

*P* value < 0.05 was considered statistically significant

## Discussion

The aim of this study was to investigate the frequency of CFTR mutations among CBAVD patients in South China and the impact of CFTR mutations on the outcomes of TESA-ICSI. By conducting whole-exome sequencing on 42 CBAVD patients, we found that 30 cases (71.4%) carried at least one CFTR gene variation, indicating a significant increase in the proportion of CFTR mutations among CBAVD patients. Moreover, comparing 30 CBAVD patients with CFTR gene mutations and 112 non-CBAVD obstructive azoospermia patients undergoing ICSI with testicular sperm extraction, we observed that although the 2PN rate was significantly higher in non-CBAVD obstructive azoospermia, there were no significant differences in fertilization rates, pregnancy rates, miscarriage rates, and live birth rates. Furthermore, by comparing the outcomes of ICSI with testicular sperm extraction between 30 CBAVD patients with CFTR mutations and 11 CBAVD patients without CFTR mutations, we found no significant differences in fertilization rate, 2PN rate, number of usable embryos, pregnancy rate, miscarriage rate, and live birth rate.

Research both domestically and internationally shows that the CFTR mutation spectrum varies significantly among different races and populations, with over 2,000 mutations reported to date [17, 21, 22]. The main types of mutations include codon deletions, splice mutations, missense mutations, non-coding region mutations, frameshift mutations, and nonsense mutations. Among these, the F508del codon mutation is the most common type in Caucasians, showing significant racial differences, with a mutation frequency of up to 70% in Caucasians but is rarely reported in China [8, 23], possibly due to it being a severe mutation with a high occurrence rate in CF, while the incidence rate of CF in China is quite low [17, 24]. Our study did not find any patients with the F508del mutation, nor did we identify any cases of CFTR codon deletion. Splice mutations are most commonly found at the junction of intron 9 and exon 10 with TG repeats and polyT mutations. The study found that the proportion of polyT polymorphisms varies greatly among different countries and populations, currently reported to be between 13% and 43.7%. The 5 T mutation is the most common mutation type among Chinese CBAVD patients [24], with a frequency ranging from 29.35% to 55.26% in Chinese patients [2, 15, 17, 25]. Our study found a 5 T mutation frequency of 50% (18/36). Additionally, research has found that the combination of the 5 T mutation with adjacent TG repeat sequence changes can affect the pathogenicity of 5 T, with shorter poly T and more TG repeats increasing the probability of CFTR gene exon 10 skipping deletion, which is involved in encoding 60 amino acids of the CFTR protein's NBD1. The absence of these amino acids would result in the loss of Cl- channel function, ultimately leading to CF and CF-related diseases [26]. Our study identified four cases of CFTR gene c.1210-12 T [5]/c.1210-34TG mutation, accounting for 11.11% (4/36). Missense mutations are most frequently found in exons 4, 7, 11, 17, and 20, and aside from F508del, are the most common and numerous mutation type in Caucasians, with R117H being the most common. However, our cases did not reveal any mutations at this site. Current research indicates that the most common mutations among Chinese CBAVD patients are 1556 V, G970A, and Q1352H [2, 27]. Our cases did not find the 1556 V mutation, which may be related to our small sample size or regional differences within China. We identified three cases each carrying the Q1352H mutation and the G970A mutation, suggesting they might be the most common missense variants among Chinese CBAVD patients.

ADGRG2 is considered the second most common mutation gene leading to CBAVD, located at Xp22.13 with 29 exons, producing 10 transcripts, with the longest transcript being 3.1 kb, encoding the adhesion G protein-coupled receptor G2. It is primarily expressed at the apical membrane of the non-ciliated epithelial cells in the human vas deferens [28]. Research has found that some CBAVD patients negative for CFTR mutations carry ADGRG2 mutations, which are considered related to the occurrence of CBAVD [10, 27–29]. Studies by Zhang et al. found high expression of ADGRG2 in the proximal epididymis and vas deferens [30]. Additionally, studies have shown that in cases of ADGRG2 mutations, proximal epididymal tissue lacks ADGRG2 protein expression, further indicating that the loss of ADGRG2 protein function due to ADGRG2 mutations is closely related to the occurrence of CBAVD [31]. In our study, we also identified a novel ADGRG2 p.Arg158His mutation among 42 CBAVD patients.

This study conducted WES on 42 Chinese CBAVD patients and found that CBAVD is primarily caused by CFTR mutations and is also associated with ADGRG2 mutations. These results are highly consistent with other WES studies on Chinese CBAVD patients [14, 15, 17]. Therefore, targeted gene testing for CFTR and ADGRG2 is more suitable for CBAVD patients compared to WES.Considering that approximately 30% of CBAVD patients have unknown genetic factors, and WES cannot cover the entire genome, whereas massive parallel sequencing, particularly whole-genome sequencing (WGS), can cover the entire genome. This allows it to detect more potential pathogenic variants, including those in regions not covered by WES. it is recommended to Massive parallel sequencing for patients whose initial screening does not identify the cause. This approach not only helps discover new pathogenic genes and mutations but also provides a more comprehensive basis for genetic counseling and personalized treatment.

CFTR mutations are the most common cause of congenital obstructive azoospermia (OA) in patients. In addition to this, current research suggests that CFTR mutations may also lead to decreased sperm vitality and fertilizing capacity, as well as reduced spermatogenic function. However, there is no clear consensus on whether CFTR mutations affect the outcomes of intracytoplasmic sperm injection (ICSI). Studies have shown that patients with cystic fibrosis (CF) have lower sperm vitality and fewer sperm compared to patients with only the CBAVD phenotype, and ICSI outcomes indicate a significantly reduced fertilization rate for CF patients [12]. Research by Lu et al. suggests that CBAVD patients have a higher miscarriage rate and significantly lower live birth rate compared to non-CBAVD patients, with similar results observed between the CFTR mutation carrier group and the non-CFTR mutation group [13]. Meanwhile, other studies report no significant differences in fertilization rates, pregnancy rates, and live birth rates between CBAVD patients carrying CFTR mutations and those not carrying mutations [13]. Wang et al.'s research shows that OA patients carrying two CFTR mutations, compared to other OA patients undergoing ICSI, found no statistical difference in either laboratory or clinical outcomes [15]. Our study first compared the ICSI outcomes of CBAVD patients with CFTR mutations to those of non-CBAVD OA patients. Although non-CBAVD OA patients may also carry CFTR mutations, literature reports a lower carrier frequency of CFTR mutations in the Chinese population [18]. This grouping can, to some extent, reveal whether CFTR mutations affect ICSI outcomes. Ultimately, our study confirmed that there were no significant differences between the two groups in terms of fertilization rate, number of usable embryos, pregnancy rate, miscarriage rate, and live birth rate.Meanwhile, to further illustrate the impact of the CFTR gene on ICSI outcomes, we compared the ICSI outcomes of 30 CBAVD patients with CFTR mutations to those of 11 CBAVD patients without CFTR mutations. We basically obtained the same conclusions. Therefore, Our study indicates that TESA-ICSI is a reliable method for achieving parenthood in both CBAVD patients and non-CBAVD OA patients, and CFTR mutations do not affect the final clinical outcomes of ICSI.

Our study has certain limitations; firstly, it is a single-center retrospective study, and the conclusions may be influenced by the small sample size and regional characteristics. Secondly, we did not screen non-CBAVD OA patients for CFTR mutations. Although the carriage rate of CFTR mutations is low in the Chinese population, this could still affect our final conclusions. Therefore, there is an urgent need for larger-scale, multicenter studies.

In summary, the genetic heterogeneity of Chinese CBAVD patients differs from the hotspot mutation pattern of Caucasians.We recommend targeted gene testing for the CFTR and ADGRG2 genes in CBAVD patients. For patients who test negative for CFTR and ADGRG2, we suggest conducting massive parallel sequencing. Based on the test results of the patients and their partners, consideration should then be given to whether to proceed with ICSI or PGS.. Additionally, although our study indicates that CFTR mutations do not affect the final ICSI clinical outcomes, considering that no pathogenic variants were found in some CBAVD patients, there are other unknown genetic factors that require further research. Genetic counseling for CBAVD patients before ART treatment is advised.

## Data Availability

Data is provided within the manuscript or supplementary information files.

## Abbreviations

CBAVD: Congenital Bilateral Aplasia of the Vas Deferens
CFTR: Cystic Fibrosis Transmembrane Conductance Regulator
ADGRG2: Adhesion G Protein-Coupled Receptor G2
ICSI: Intracytoplasmic Sperm Injection
TESA: Testicular Sperm Aspiration
ART: Assisted Reproductive Technology
WES: Whole Exome Sequencing
gDNA: Genomic DNA
BQSR: Base Quality Score Recalibration
VQSR: Variant Quality Score Recalibration
HGMD: Human Gene Mutation Database
OA: Obstructive Azoospermia
